# Correction: Detecting the impact of temperature on transmission of Zika, dengue, and chikungunya using mechanistic models

**DOI:** 10.1371/journal.pntd.0010514

**Published:** 2022-06-02

**Authors:** Erin A. Mordecai, Jeremy M. Cohen, Michelle V. Evans, Prithvi Gudapati, Leah R. Johnson, Catherine A. Lippi, Kerri Miazgowicz, Courtney C. Murdock, Jason R. Rohr, Sadie J. Ryan, Van Savage, Marta S. Shocket, Anna Stewart Ibarra, Matthew B. Thomas, Daniel P. Weikel

S1 Text contains errors in SOM1 Figs L and M and the second paragraph of the Supplementary Results section. Please see the corrected S1 Text file here.

## Supporting information

S1 Text Supplementary Results, References, and Figures A-O. (PDF)
