# Supplementary material for: Detecting the impact of temperature on transmission of Zika, dengue, and chikungunya using mechanistic models
Source: PLoS Negl Trop Dis. 2017 Apr 27;11(4):e0005568. doi: 10.1371/journal.pntd.0005568 (PMC5423694; doi:10.1371/journal.pntd.0005568)
Supplement: S2 Text — (PDF) [file pntd.0005568.s002.pdf]

Supporting Information: S2 Text

Detecting the impact of temperature on transmission of Zika, dengue and chikungunya using mechanistic models

Erin A. Mordecai, Jeremy M. Cohen, Michelle V. Evans, Prithvi Gudapati, Leah R. Johnson, Catherine Lippi, Kerri Miazgowicz, Courtney C. Murdock, Jason R. Rohr, Sadie J. Ryan, Van Savage, Marta S. Shocket, Anna Stewart Ibarra, Matthew B. Thomas, Daniel P. Weikel
correspondence to: emordeca@stanford.edu

This PDF file includes:

Tables A-D

Table A. Data used in the *Ae. albopictus* R_0 model. Each trait parameter symbol, definition, data sources, and thermal response function (Quad = quadratic) are shown on the left. Mean and 95% credible interval (95% HPD interval) for the critical thermal minimum (T_0), maximum, (T_m), and a rate constant (c) are given for each trait in the three right sections. * indicates unpublished data provided by Francis Ezeakacha.

Trait	Definition	Refs	Function	T_0 Mean	T_0 95% CI		T_m Mean	T_m 95% CI		c Mean	c 95% CI	
a	biting rate, calculated as reciprocal of oviposition cycle length (1/days)	(8)	Brière	10.25	5.84	14.82	38.32	36.60	40.51	1.93E-04	1.27E-04	2.61E-04
TFD	eggs laid per female per gonotrophic cycle (number/female)	(8,9)	Brière	8.02	3.18	13.08	35.65	35.00	36.51	4.88E-02	3.21E-02	6.72E-02
pEA	mosquito egg-to-adult survival probability	(8,10–13)	Quad	9.04	6.37	11.67	39.33	37.17	41.62	-3.61E-03	-4.74E-03	-2.59E-03
MDR	mosquito egg-to-adult development rate (1/days)	(8–11,13–17)*	Brière	8.60	4.43	12.29	39.66	37.78	41.70	6.38E-05	4.67E-05	8.23E-05
lf	mosquito adult lifespan (days)	(9,14,18)	Quad	13.41	10.53	16.11	31.51	29.14	33.57	-1.43E+00	-2.16E+00	-6.89E-01
b	probability that a mosquito infected with DENV becomes infectious (has virus in the salivary glands)	(19)	Brière	15.84	11.42	19.87	36.40	36.00	36.93	7.35E-04	4.36E-04	1.04E-03
c	probability that a mosquito fed on DENV-infected blood becomes infected	(19)	Brière	3.62	0.00	9.90	36.82	36.00	37.88	4.39E-04	3.29E-04	5.66E-04
PDR	DENV extrinsic incubation rate (reciprocal of the extrinsic incubation period: the time required	(19)	Brière	10.39	2.82	17.60	43.05	37.54	49.56	1.09E-04	5.45E-05	1.76E-04

for an exposed mosquito to become infectious; 1/days)			

Table B.

Data used in the *Ae. aegypti* R_0 model. Each trait parameter symbol, definition, data sources, and thermal response function (Quad = quadratic) are shown on the left. Mean and 95% credible interval (95% HPD interval) for the critical thermal minimum (T_0), maximum, (T_m), and a rate constant (c) are given for each trait in the three right sections.

Trait	Definition	Refs	Function	T_0 Mean	T_0 95% CI		T_m Mean	T_m 95% CI		c Mean	c 95% CI	
a	biting rate, calculated as reciprocal of oviposition cycle length (1/days)	(1,20)	Brière	13.35	8.27	17.41	40.08	40.00	40.28	2.02E-04	1.20E-04	2.80E-04
EFD	eggs laid per female per day (number/female/day)	(21, 22)	Brière	14.58	8.08	20.60	34.61	34.00	35.77	8.56E-03	3.78E-03	1.41E-02
pEA	mosquito egg-to-adult survival probability	(10, 23–26)	Quad	13.56	12.56	14.51	38.29	37.54	39.02	-5.99E-03	-6.82E-03	-5.13E-03
MDR	mosquito egg-to-adult development rate (1/days)	(10, 23–27)	Brière	11.36	7.19	15.03	39.17	39.00	39.54	7.86E-05	5.75E-05	9.93E-05
lf	mosquito adult lifespan (days)	(21, 22)	Quad	9.16	6.69	12.33	37.73	35.68	39.89	-1.48E-01	-2.06E-01	-9.77E-02
b	probability that a mosquito infected with DENV becomes infectious (has virus in the salivary glands)	(28–30)	Brière	17.05	12.56	21.26	35.83	35.06	36.69	8.49E-04	5.07E-04	1.20E-03
c	probability that a mosquito fed on DENV-infected blood becomes infected	(28, 30)	Brière	12.22	5.61	17.76	37.46	35.70	39.29	4.91E-04	3.33E-04	6.41E-04
PDR	DENV extrinsic incubation rate (reciprocal of the extrinsic incubation period: the time required for an exposed mosquito to become infectious; 1/days)	(28, 30–34)	Brière	10.68	3.86	18.33	45.90	39.73	52.92	6.65E-05	3.60E-05	1.09E-04

Table C.

Aedes spp. trait thermal response data used to generate informative priors for the main *Ae. albopictus* and *Ae. aegypti* R_0 models. Mean and 95% credible interval (95% HPD interval) for the critical thermal minimum (T_0), maximum, (T_m), and a rate constant (c) are given for each trait in the three right sections.

Trait	Definition	Refs	Function	T_0			T_m			c		
				Mean	95% CI		Mean	95% CI		Mean	95% CI	
a	biting rate, calculated as reciprocal of oviposition cycle length (1/days)	(18)	Brière	14.67	10.67	18.34	41.00	37.56	44.99	2.71E-04	1.59E-04	4.09E-04
EFD	eggs laid per female per day (number/female/day)	(35)	Brière	14.06	11.32	16.60	32.03	30.95	33.35	2.08E-02	1.36E-02	2.89E-02
pEA	mosquito egg-to-adult survival probability	(36)	Quad	7.68	6.48	8.90	38.31	36.99	39.57	-3.36E-03	-4.02E-03	-2.72E-03
MDR	mosquito egg-to-adult development rate (1/days)	(35, 36)	Brière	15.12	9.56	19.93	37.67	36.54	38.45	1.49E-04	8.59E-05	2.17E-04
lf	mosquito adult lifespan (days)	(35)	Quad	16.63	15.93	17.25	31.85	31.16	32.64	-1.24E+00	-1.50E+00	-9.76E-01
b	probability that a mosquito infected with flavivirus becomes infectious (has virus in the salivary glands)	(37)	Brière	12.05	8.18	15.09	32.79	32.02	34.32	9.86E-04	5.97E-04	1.34E-03
c	probability that a mosquito fed on flavivirus-infected blood becomes infected	(37)	Brière	1.51	0.00	4.11	34.74	32.87	37.18	5.23E-04	4.10E-04	6.32E-04
PDR	WNV, SLEV, WEEV extrinsic incubation rate (reciprocal of the extrinsic incubation period: the time required for an exposed mosquito to become infectious; 1/days)	(38)	Brière	11.50	3.43	18.55	38.97	33.08	45.00	1.04E-04	3.79E-05	1.93E-04

Table D.

Generalized linear models used to validate the $R_0(T)$ model. Presence/absence models used a Binomial distribution and incidence models used a Gamma distribution. "- DEN" indicates that separate y-intercepts were not fit for DENV versus CHIKV/ZIKV (only interaction terms). Abbreviations used in the table are listed below.

Presence/Absence Models

model	formula	BIC	Model prob.	D^2	OOS model prob.	OOS classification error (DENV)	OOS classification error (CHIKV & ZIKV)
PA1	pa ~ lpop*DEN - DEN	1444	0	0.382	0	0.224	0.309
PA2	pa ~ (propTGDP + lGDP +lpop)*DEN - DEN	1644	0	0.469	0	0.142	0.434
PA3	pa ~ (R0.GR0+lpop)*DEN - DEN	1465	0	0.461	0	0.093	0.338
PA4	pa ~ (R0.GR0 + propTGDP+ lGDP+lpop)*DEN - DEN	1375	0.008	0.505	0.01	0.118	0.342
PA5	pa ~ R0.GR0 + propTGDP + (lGDP+lpop)*DEN - DEN	1365	0.992	0.503	0.99	0.12	0.351
PA6	pa ~ R0.GR0.alt*DEN - DEN	1455	0	0.432	0	0.096	0.337

Incidence Models

model	formula	BIC	Model prob.	D^2	OOS model prob.	OOS mean absolute percentage error
IM1	l.inc ~ lpop*DEN-DEN	2593	0	0.465	0	0.169
IM2	l.inc ~ (propTGDP + lGDP +lpop)*DEN-DEN	2559	0	0.506	0	0.169
IM3	l.inc ~ (R0 +lpop)*DEN-DEN	2484	0	0.55	0	0.148
IM4	l.inc ~ (lR0.alt)*DEN-DEN	2433	0.052	0.595	0.058	0.14
IM5	l.inc ~ (R0 + propTGDP + lGDP + lpop)*DEN-DEN	2427	0.948	0.595	0.942	0.14
IM6	l.inc ~ (R0 + propTGDP + lpop)*DEN + lGDP -DEN	2501	0	0.531	0	0.153

Model terms and abbreviations

OOS = out-of-sample

BIC = Bayesian Information Criterion

Model prob. = model probability based on BIC

D^2 = deviance explained

pa = presence-absence

lpop = log(population size)

DEN = indicator of DENV (DEN = 1) or CHIKV or ZIKV (DEN = 0); -DEN indicates that the main effect for this term was not included

lGDP = log(gross domestic product)

propTGDP = proportion of GDP in tourism

R0.GR0 = Prob($R_0(T) > 0$)

R0.GR0.alt = Prob($R_0(T) > 0$) * log(population size)

l.inc = log(incidence) for incidence > 0

R0 = $R_0(T)$

lR0.alt = log($R_0(T)$ * population size)
