# Supplementary material for: Correction: Detecting the impact of temperature on transmission of Zika, dengue, and chikungunya using mechanistic models
Source: PLoS Negl Trop Dis. 2022 Jun 2;16(6):e0010514. doi: 10.1371/journal.pntd.0010514 (PMC9162309; doi:10.1371/journal.pntd.0010514)
Supplement: S1 Text — (PDF) [file pntd.0010514.s001.pdf]

Supporting Information: S1 Text

Detecting the impact of temperature on transmission of Zika, dengue and chikungunya using mechanistic models

Erin A. Mordecai, Jeremy M. Cohen, Michelle V. Evans, Prithvi Gudapati, Leah R. Johnson, Catherine Lippi, Kerri Miazgowicz, Courtney C. Murdock, Jason R. Rohr, Sadie J. Ryan, Van Savage, Marta S. Shocket, Anna Stewart Ibarra, Matthew B. Thomas, Daniel P. Weikel
correspondence to: emordeca@stanford.edu

This PDF file includes:

Supplementary Results
References
Figs. A-O

Supplementary Results

Sensitivity analyses

We explored several methods of sensitivity analyses to examine how model predictions change with different thermal response assumptions. Because no temperature-sensitive vector competence or extrinsic incubation period (EIP) data were available for CHIKV or ZIKV, we were particularly interested in the R_0 model sensitivity to the thermal responses for these traits. We explored the impact of changes in *b, c,* and *PDR* by calculating R_0 for all posterior parameter samples with those focal traits shifted in the following ways: entire curves shifted ±3°C and ±5°C for all three traits, entire curves shifted ±3°C and ±5°C for each trait individually, and curves made 3°C wider or narrower without changing the mean for all three traits. We examined the impact of each modification on the thermal minimum, maximum, and optimum (T_0, T_m, and T_{pk}) for R_0. For *Ae. albopictus*, all shifts in trait thermal responses shifted T_{pk} by < 1°C, T_0 by approximately the amount of the trait shift (e.g., +3°C for the models with the traits shifted by +3°C), and had little effect on T_m (Fig. J in S1 Text). Similarly, for *Ae. aegypti* all models shifted T_{pk} by < 2°C, T_0 by less than or equal to the amount of the trait shift, and had little effect on T_m, with the exception of the -5°C trait shift, which reduced T_m by 5°C (Fig. K in S1 Text). These analyses indicate that the optimal and maximum temperatures for transmission are robust to error in the vector competence and EIP thermal responses. By contrast, the minimum temperature for transmission may be sensitive to these trait thermal responses, so it is important to experimentally measure vector competence and EIP, particularly at low temperatures, for each mosquito and pathogen species pair of interest.

We also used sensitivity analyses to characterize the degree to which the temperature response of each individual trait drives the overall temperature response of R_0 (i.e., $(1/R_0)(dR_0/dX)$ for each parameter X). For both the *Ae. aegypti* and the *Ae. albopictus* models, we found that the parasite development rate (PDR) thermal response strongly increased and the lifespan (lf) thermal response strongly decreased the response of R_0 to temperature (Figs. L-M in S1 Text).

We were interested in which trait's thermal response was driving the difference in optimal temperature for *Ae. aegypti* versus *Ae. albopictus* transmission. To investigate this, we sequentially swapped thermal responses from one model to the other (e.g., calculated R_0 with all *Ae. albopictus* trait thermal responses except one from *Ae. aegypti* and vice versa). Mosquito lifespan was the key driver in the difference between the two R_0-versus-temperature models. Although the optimal temperatures for mosquito lifespan were similar, the thermal breadth was much narrower for *Ae. albopictus* than for *Ae. aegypti*. R_0 is strongly limited by short mosquito lifespans at high temperatures, where viral extrinsic incubation is very rapid, so expanding the thermal breadth for this trait has a large effect on the optimum.

Uncertainty analyses

We estimated how uncertainty in the trait thermal responses contributed to uncertainty in R_0 versus temperature. First, we calculated the width of the 95% credible interval for R_0 with all parameters sampled from their posterior distributions across temperatures. Then,

we calculated the width of the 95% credible interval for R_0 when each trait was sampled from its posterior distribution individually, while the remaining parameters were fixed at their posterior mean. We compared the width of the intervals when just one parameter was sampled from its posterior distribution to the width when all parameters were sampled to calculate the relative contribution of each parameter to uncertainty at each temperature. For *Ae. albopictus*, mosquito lifespan (*lf*) contributed most to uncertainty from 24-35°C and transmission probability (*b*), followed by infection probability (*c*), contributed most to uncertainty from 16-24°C (Fig. N in S1 Text). For *Ae. aegypti*, biting rate (*a*) contributed most to uncertainty from 29-35°C, transmission probability (*b*) contributed most to uncertainty from 13-28°C, and mosquito lifespan (*lf*), fecundity (*EFD*), and infection probability (*c*) all contributed substantially to uncertainty from 13-35°C (Fig. O in S1 Text).

References

1. Morin CW, Monaghan AJ, Hayden MH, Barrera R, Ernst K. Meteorologically driven simulations of dengue epidemics in San Juan, PR. PLoS Negl Trop Dis. 2015 Aug 14;9(8):e0004002.

2. Wesolowski A, Qureshi T, Boni MF, Sundsøy PR, Johansson MA, Rasheed SB, et al. Impact of human mobility on the emergence of dengue epidemics in Pakistan. Proc Natl Acad Sci. 2015 Sep 8;201504964.

3. Liu-Helmersson J, Stenlund H, Wilder-Smith A, Rocklöv J. Vectorial Capacity of Aedes aegypti: Effects of Temperature and Implications for Global Dengue Epidemic Potential. PLoS ONE. 2014 Mar 6;9(3):e89783.

4. Johansson MA, Powers AM, Pesik N, Cohen NJ, Staples JE. Nowcasting the spread of chikungunya virus in the Americas. PLoS ONE. 2014 Aug 11;9(8):e104915.

5. Caminade C, Turner J, Metelmann S, Hesson JC, Blagrove MSC, Solomon T, et al. Global risk model for vector-borne transmission of Zika virus reveals the role of El Niño 2015. Proc Natl Acad Sci. 2017 Jan 3;114(1):119–24.

6. Zhang Q, Sun K, Chinazzi M, Pastore-Piontti A, Dean NE, Rojas DP, et al. Projected spread of Zika virus in the Americas. bioRxiv. 2016 Dec 12;066456.

7. Dunn PK, Smyth GK. Randomized quantile residuals. J Comput Graph Stat. 1996;5.3:236–44.

8. Delatte H, Gimonneau G, Triboire A, Fontenille D. Influence of temperature on immature development, survival, longevity, fecundity, and gonotrophic cycles of Aedes albopictus, vector of chikungunya and dengue in the Indian Ocean. J Med Entomol. 2009 Jan 1;46(1):33–41.

9. Ezeakacha N. Environmental impacts and carry-over effects in complex life cycles: the role of different life history stages. Dissertation [Internet]. 2015 Dec 11; Available from: http://aquila.usm.edu/dissertations/190

10. Westbrook CJ. Larval ecology and adult vector competence of invasive mosquitoes Aedes albopictus and Aedes aegypti for Chikungunya virus [Internet]. University of Florida; 2010 [cited 2013 Oct 23]. Available from: http://etd.fcla.edu/UF/UFE0041830/westbrook_c.pdf

11. Wiwatanaratanabutr S, Kittayapong P. Effects of temephos and temperature on Wolbachia load and life history traits of Aedes albopictus. Med Vet Entomol. 2006;20(3):300–307.

12. Teng H-J, Apperson CS. Development and Survival of Immature Aedes albopictus and Aedes triseriatus (Diptera: Culicidae) in the Laboratory: Effects of Density, Food, and Competition on Response to Temperature. J Med Entomol. 2000 Jan 1;37(1):40–52.

13. Muturi EJ, Lampman R, Costanzo K, Alto BW. Effect of temperature and insecticide stress on life-history traits of Culex restuans and Aedes albopictus (Diptera: Culicidae). J Med Entomol. 2011 Mar;48(2):243–50.

14. Alto BW, Juliano SA. Temperature effects on the dynamics of Aedes albopictus (Diptera: Culicidae) populations in the laboratory. J Med Entomol. 2001 Jul;38(4):548–56.

15. Westbrook CJ, Reiskind MH, Pesko KN, Greene KE, Lounibos LP. Larval environmental temperature and the susceptibility of Aedes albopictus Skuse (Diptera: Culicidae) to chikungunya virus. Vector-Borne Zoonotic Dis. 2010 Apr 28;10(3):241–7.

16. Briegel H, Timmermann SE. Aedes albopictus (Diptera: Culicidae): Physiological aspects of development and reproduction. J Med Entomol. 2001 Jul 1;38(4):566–71.

17. Calado DC, Silva MAN da. Avaliação da influência da temperatura sobre o desenvolvimento de Aedes albopictus. Rev Saúde Pública. 2002 Apr;36(2):173–9.

18. Calado DC, Navarro-Silva MA. Influência da temperatura sobre a longevidade, fecundidade e atividade hematofágica de Aedes (Stegomyia) albopictus Skuse, 1894 (Diptera, Culicidae) sob condições de laboratório. Rev Bras Entomol. 2002;46(1):93–8.

19. Xiao F-Z, Zhang Y, Deng Y-Q, He S, Xie H-G, Zhou X-N, et al. The effect of temperature on the extrinsic incubation period and infection rate of dengue virus serotype 2 infection in Aedes albopictus. Arch Virol. 2014 Jul 3;159(11):3053–7.

20. Focks DA, Haile DG, Daniels E, Mount GA. Dynamic life table model for Aedes aegypti (Diptera: Culicidae): analysis of the literature and model development. J Med Entomol. 1993 Nov;30(6):1003–17.

21. Yang HM, Macoris MLG, Galvani KC, Andrighetti MTM, Wanderley DMV. Assessing the effects of temperature on the population of Aedes aegypti, the vector of dengue. Epidemiol Infect. 2009;137(08):1188–202.

22. Beserra EB, Fernandes CRM, Silva SA de O, Silva LA da, Santos JW dos. Efeitos da temperatura no ciclo de vida, exigências térmicas e estimativas do número de gerações anuais de Aedes aegypti (Diptera, Culicidae). Iheringia Sér Zool [Internet]. 2009 [cited 2015 Sep 10]; Available from: http://agris.fao.org/agris-search/search.do?recordID=XS2010500501

23. Rueda L, Patel K, Axtell R, Stinner R. Temperature-dependent development and survival rates of Culex quinquefasciatus and Aedes aegypti (Diptera: Culicidae). J Med Entomol. 1990;27(5):892–8.

24. Tun-Lin W, Burkot T, Kay B. Effects of temperature and larval diet on development rates and survival of the dengue vector Aedes aegypti in north Queensland, Australia. Med Vet Entomol. 2000;14(1):31–7.

25. Kamimura K, Matsuse IT, Takahashi H, Komukai J, Fukuda T, Suzuki K, et al. Effect of temperature on the development of Aedes aegypti and Aedes albopictus. Med Entomol Zool. 2002;53(1):53–8.

26. Eisen L, Monaghan AJ, Lozano-Fuentes S, Steinhoff DF, Hayden MH, Bieringer PE. The impact of temperature on the bionomics of Aedes (Stegomyia) aegypti, with special reference to the cool geographic range margins. J Med Entomol. 2014 May 1;51(3):496–516.

27. Couret J, Dotson E, Benedict MQ. Temperature, Larval Diet, and Density Effects on Development Rate and Survival of Aedes aegypti (Diptera: Culicidae). PLoS ONE. 2014 Feb 3;9(2):e87468.

28. Watts DM, Burke DS, Harrison BA, Whitmire RE, Nisalak A. Effect of temperature on the vector efficiency of Aedes aegypti for dengue 2 virus. Am J Trop Med Hyg. 1987 Jan;36(1):143–52.

29. Alto BW, Bettinardi D. Temperature and dengue virus infection in mosquitoes: independent effects on the immature and adult stages. Am J Trop Med Hyg. 2013 Mar;88(3):497–505.

30. Carrington LB, Armijos MV, Lambrechts L, Scott TW. Fluctuations at a low mean temperature accelerate dengue virus transmission by Aedes aegypti. PLoS Negl Trop Dis. 2013 Apr 25;7(4):e2190.

31. Davis NC. The effect of various temperatures in modifying the extrinsic incubation period of the yellow fever virus in Aedes aegypti. Am J Epidemiol. 1932 Jul 1;16(1):163–76.

32. Focks DA, Daniels E, Haile DG, Keesling JE. A simulation model of the epidemiology of urban dengue fever: literature analysis, model development, preliminary validation, and samples of simulation results. Am J Trop Med Hyg. 1995 Nov;53(5):489–506.

33. McLean DM, Clarke AM, Coleman JC, Montalbetti CA, Skidmore AG, Walters TE, et al. Vector capability of Aedes aegypti mosquitoes for California encephalitis and dengue viruses at various temperatures. Can J Microbiol. 1974 Feb 1;20(2):255–62.

34. McLean DM, Miller MA, Grass PN. Dengue virus transmission by mosquitoes incubated at low temperatures. Mosq News [Internet]. 1975 [cited 2015 Aug 26]; Available from: http://agris.fao.org/agris-search/search.do?recordID=US19760088008

35. Joshi DS. Effect of fluctuating and constant temperatures on development, adult longevity and fecundity in the mosquito Aedes krombeini. J Therm Biol. 1996 Jun;21(3):151–4.

36. JALIL M. Effect of temperature on larval growth of Aedes triseriatus. J Econ Entomol. 1972;65(2):625–6.

37. Lambrechts L, Paaijmans KP, Fansiri T, Carrington LB, Kramer LD, Thomas MB, et al. Impact of daily temperature fluctuations on dengue virus transmission by Aedes aegypti. Proc Natl Acad Sci. 2011 May 3;108(18):7460–5.

38. Reisen WK, Fang Y, Martinez VM. Effects of temperature on the transmission of West Nile virus by Culex tarsalis (Diptera: Culicidae). J Med Entomol. 2006 Mar;43(2):309–17.

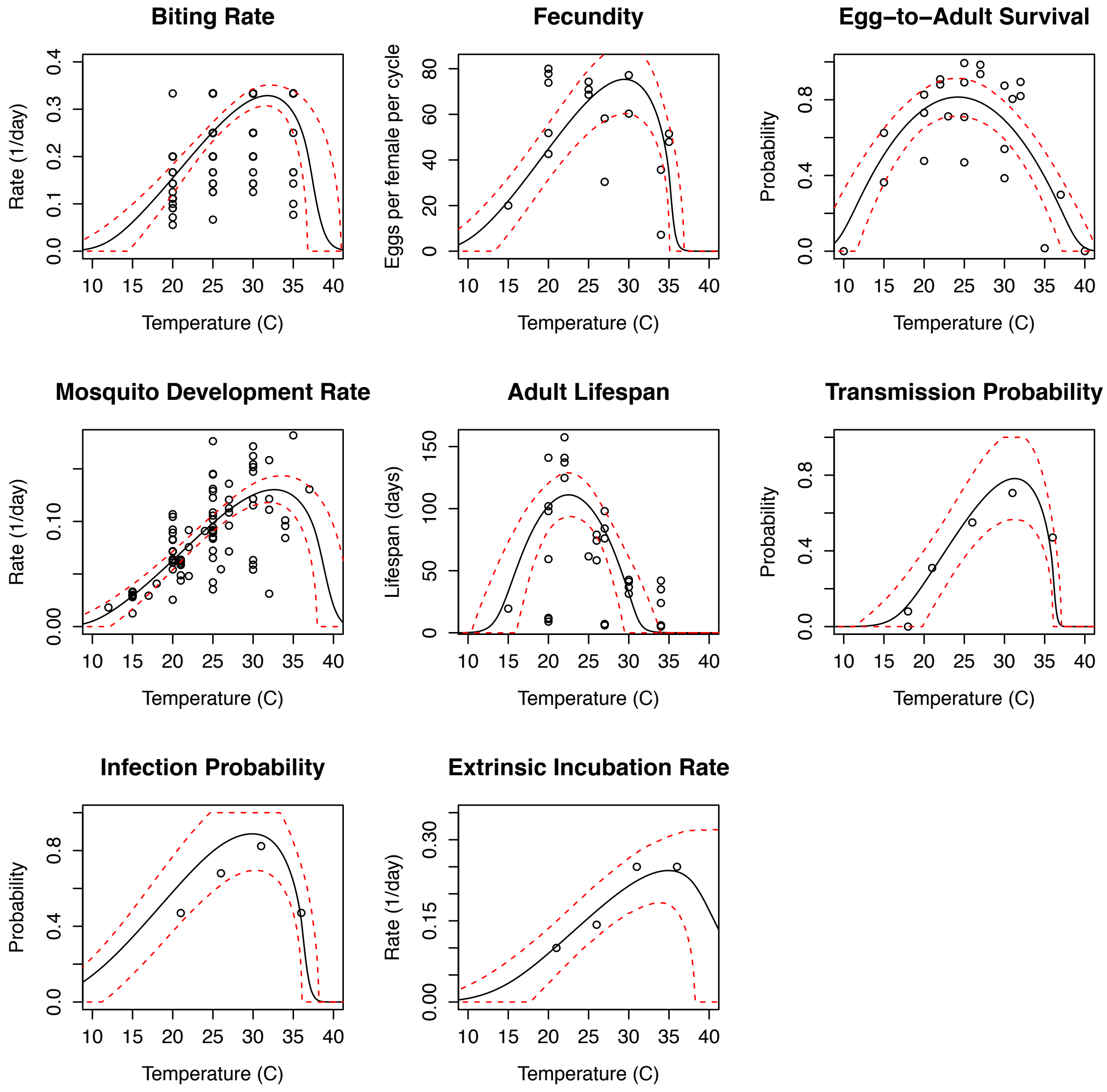

Fig. A

Thermal responses of *Ae. albopictus* and DENV traits that drive transmission (parameter names and data sources listed in Table A in S2 Text). Informative priors based on data from additional *Aedes* spp. and flavivirus studies helped to constrain uncertainty in the model fits (see Materials and Methods; Table C in S2 Text). Points are the data. Black solid lines are the mean model fits; red dashed lines are the 95% credible intervals.

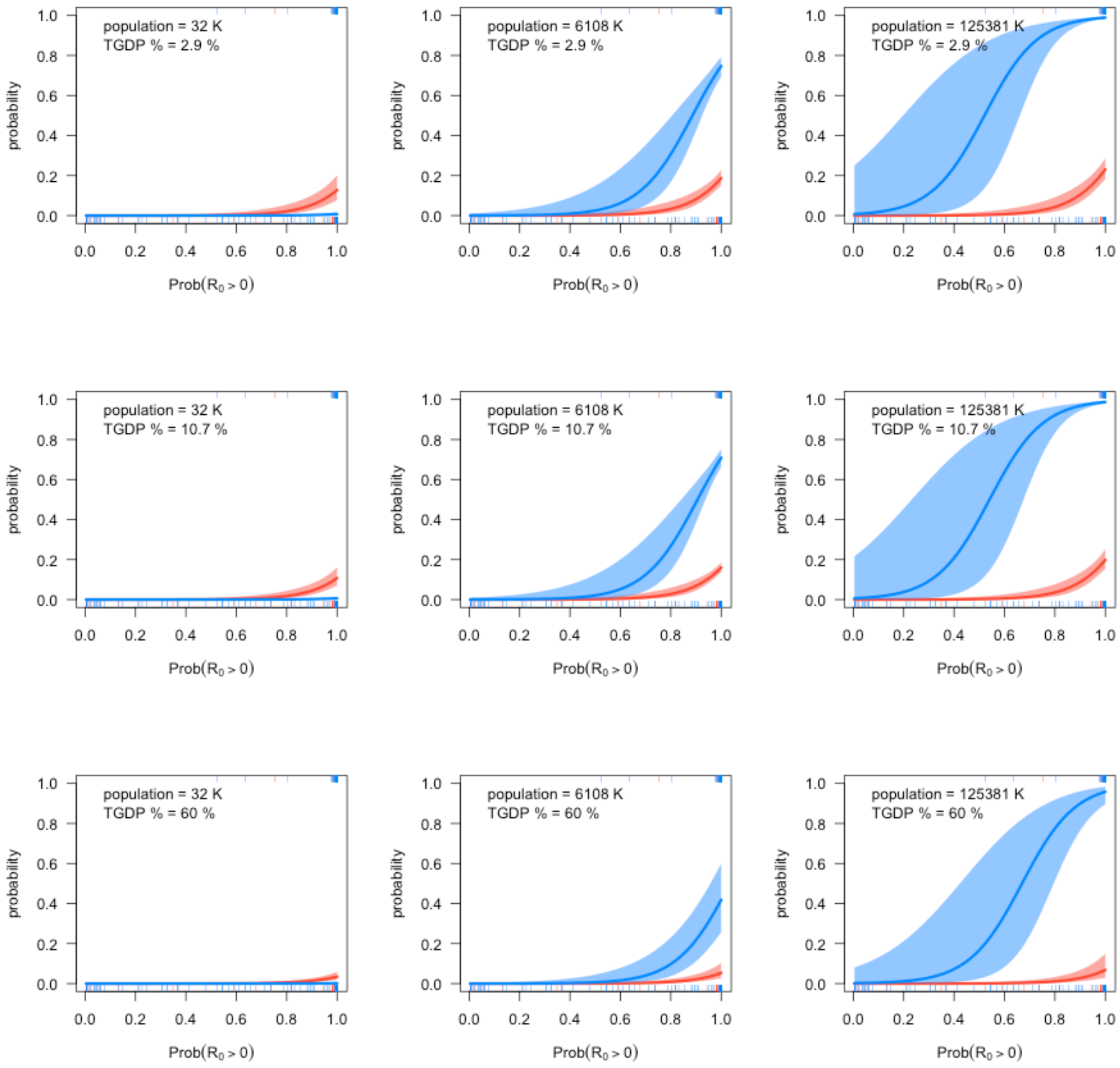

Fig. B

Plots of the probability that $R_0 > 0$ (GR_0) versus the probability of transmission predicted from presence/absence model PA5, for different levels of percent tourism in GDP (TGDP) across different rows and population size (population) across different columns, at the median value of GDP (per capita GDP = $7274). Red lines: CHIKV and ZIKV. Blue lines: DENV. Lines are the mean model fits and shaded areas are the standard errors. Tick marks show the data.

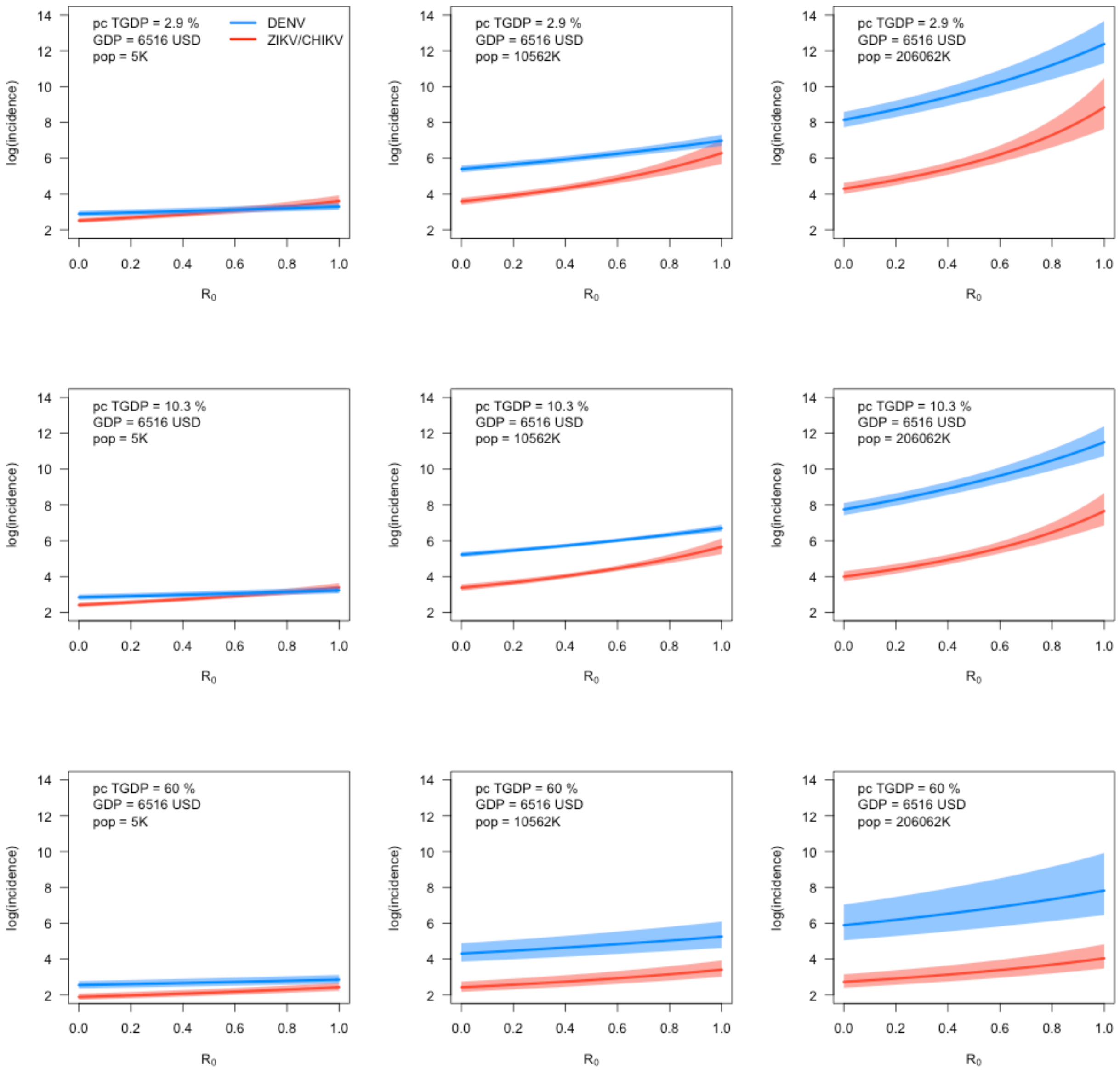

Fig. C

Plots of R_0 versus the log of incidence predicted from incidence model IM5, for different levels of percent tourism in GDP (pc TGDP) across different rows and of population size (pop) across different columns, at the median value of GDP (per capita GDP = $6516). Red lines: CHIKV and ZIKV. Blue lines: DENV. Lines are the mean model fits and shaded areas are the standard errors.

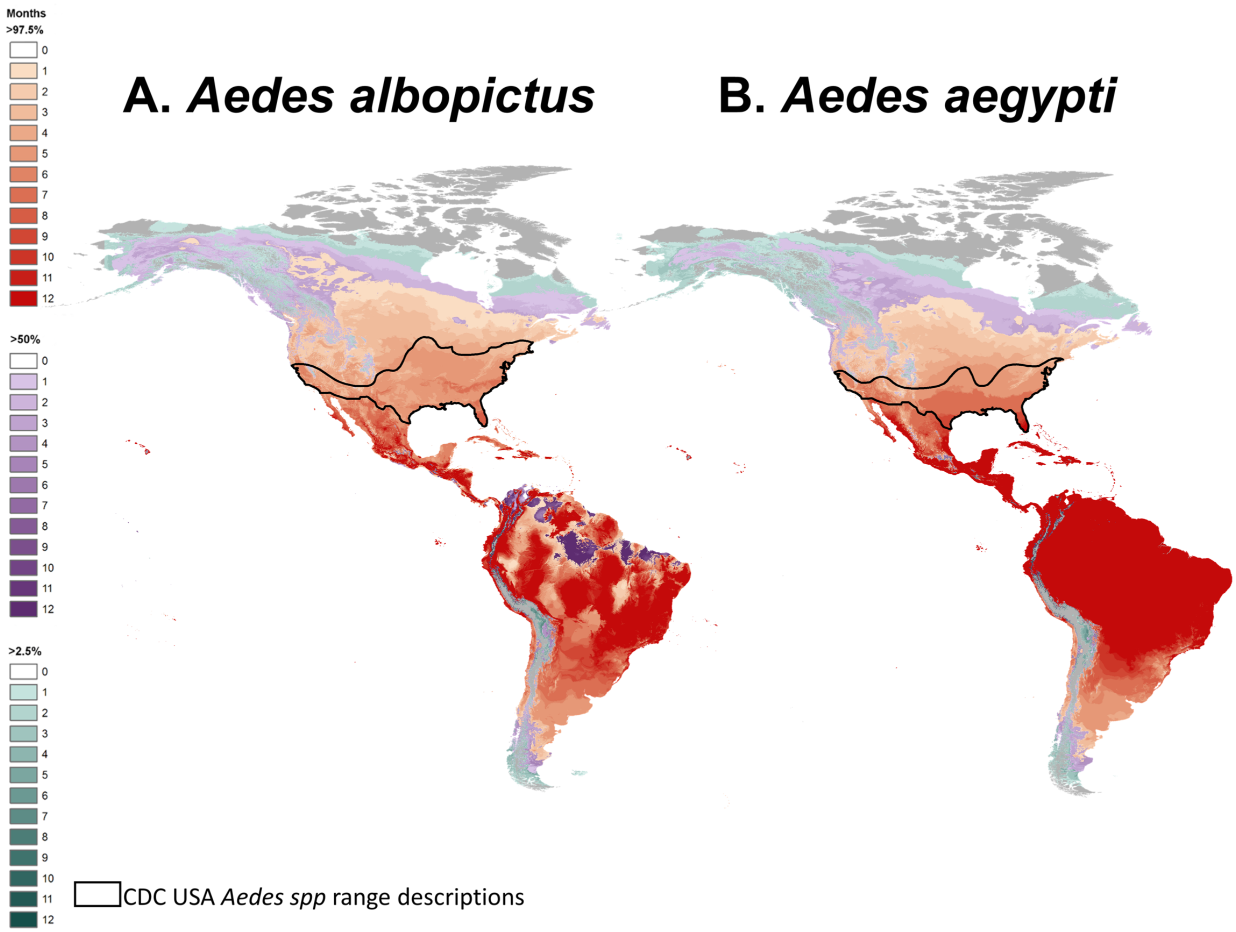

Fig. D

Map of predicted temperature suitability for virus transmission by *Ae. albopictus* (A) and *Ae. aegypti* (B). Color indicates the consecutive months in which temperature is permissive for transmission (predicted $R_0 > 0$) for *Aedes* spp. transmission. Red, minimum likely range (> 97.5% probability that $R_0 > 0$), purple, median likely range (> 50% probability that $R_0 > 0$), teal, maximum likely range (> 2.5% probability that $R_0 > 0$). Black line indicates the CDC *Aedes* spp. range estimates in the United States. Model suitability predictions combine temperature mean and 8°C daily variation and are informed by laboratory data (Fig. 1, Fig. A in S1 Text) and validated against field data (Fig. 3).

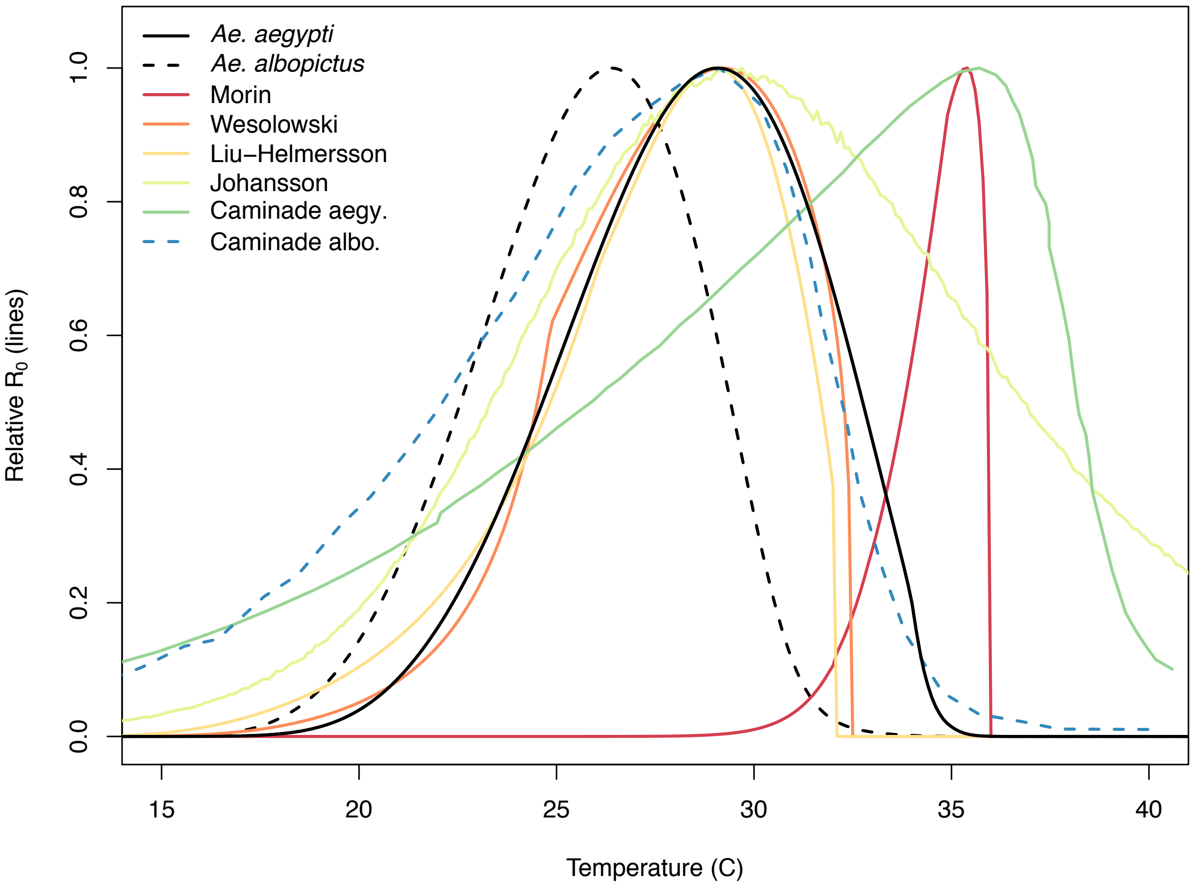

Fig. E

R_0 versus temperature models for *Ae. aegypti* (black solid line) and *Ae. albopictus* (black dashed line) and models based on the thermal responses listed in five previous studies: models of DENV transmission by *Ae. aegypti* by Morin et al. (1) (red line), Wesolowski et al. (2) (orange line), and Liu-Helmersson et al. (3) (gold line), a model of CHIKV transmission by *Ae. aegypti* by Johansson et al. (4) (light green line), and models of ZIKV transmission by *Ae. aegypti* (green line) and *Ae. albopictus* (blue dashed line) by Caminade et al. (5), which are based on DENV extrinsic incubation rate data. Note that we could not reproduce the functional forms in Caminade et al.'s Fig. S15 from the equations in their Table 1, so we digitized the $R_0(T)$ functions shown in Fig. S15D. We were unable to fully reproduce the thermal response functions from Zhang et al. (6) and their model-predicted $R_0(T)$ relationship is not shown.

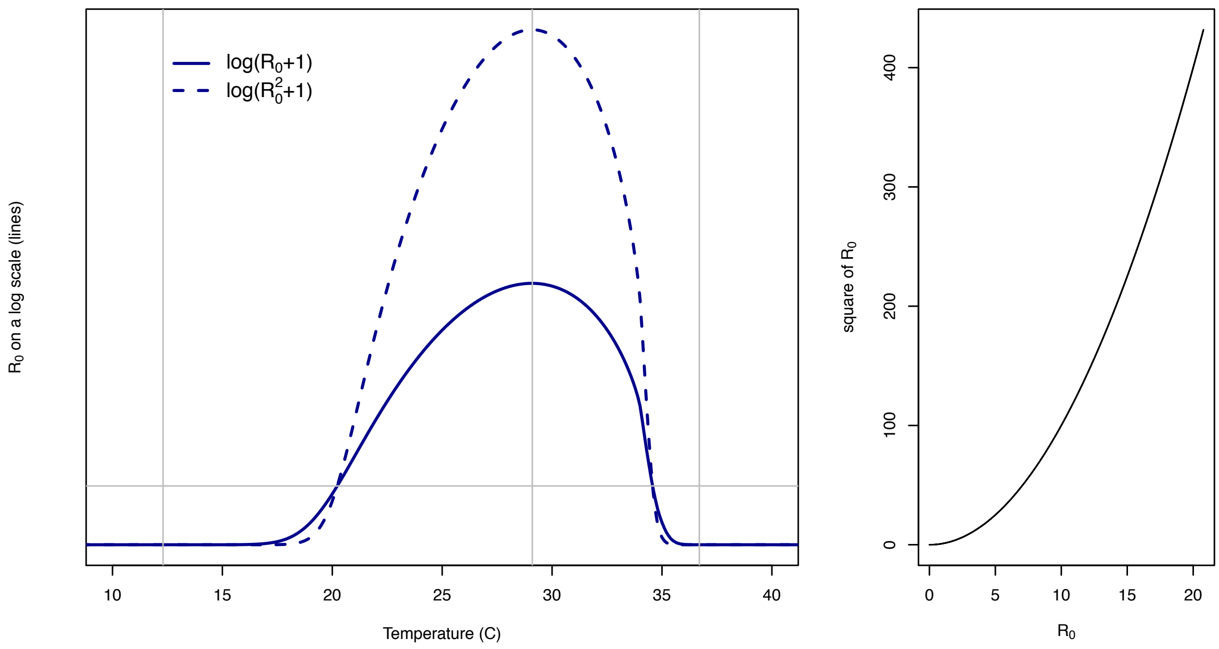

Fig. F

Comparing two commonly used formulations for R_0: equation (1), indicated as R_0, and equation (1) squared, indicated as R_0^2. Left panel: plot of R_0 and R_0^2 as functions of temperature, shown as $\log(R_0 + 1)$ (solid line) and $\log(R_0^2 + 1)$ (dashed line) for ease of comparison. Vertical lines indicate the critical thermal minimum and maximum, at which both functions equal zero, and the optimal temperature, at which both functions are maximized. Horizontal line indicates $R_0 = R_0^2 = 1$, at which points their temperature values are also equal. Right panel: monotonic relationship between R_0 and R_0^2.

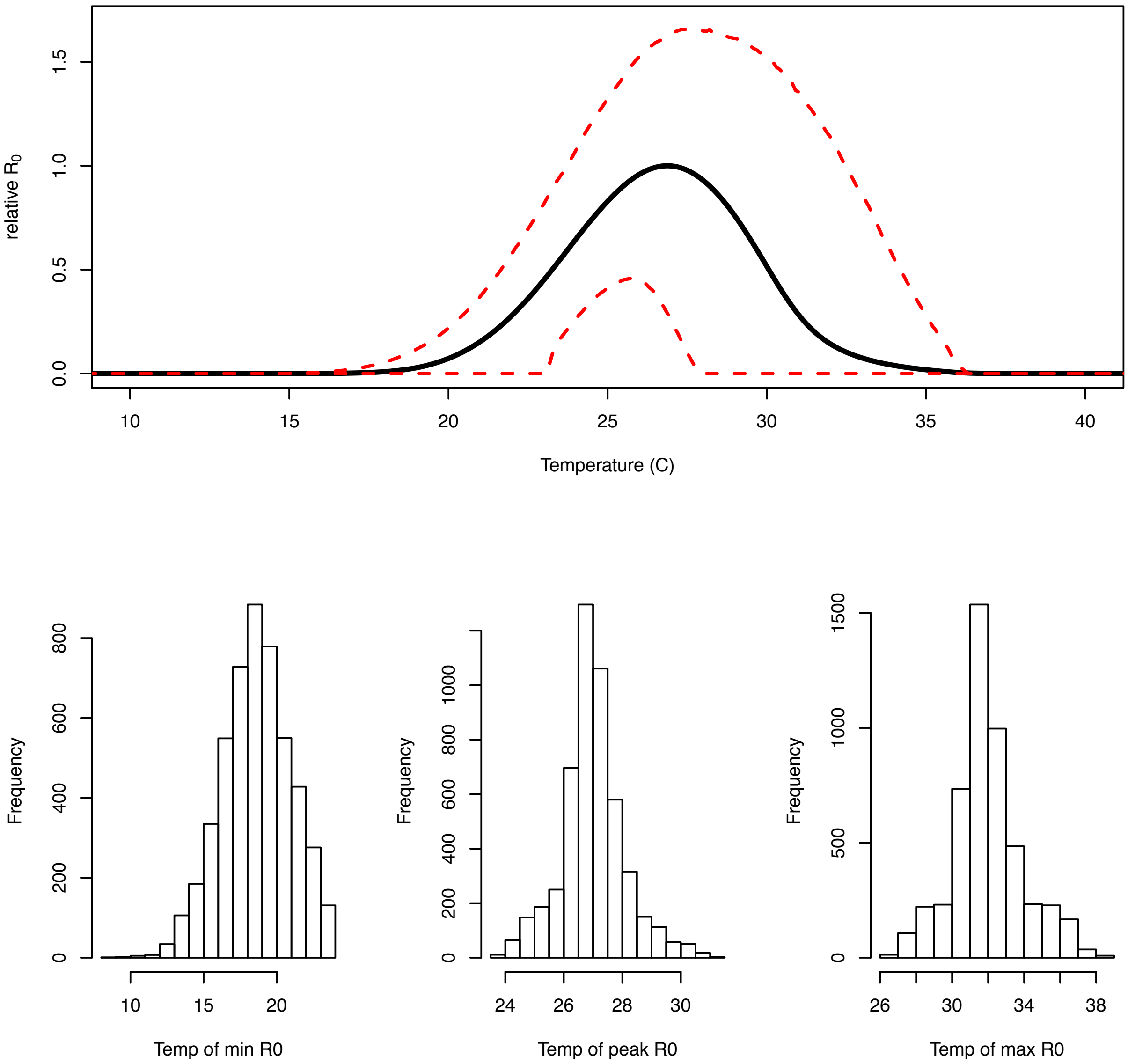

Fig. G

Top, uninformative prior model of *Ae. albopictus* R_0 versus temperature model mean (black line) and 95% highest posterior density intervals (red dashed lines), for constant temperatures. Bottom, histograms of the minimum, maximum, and optimum temperatures for transmission.

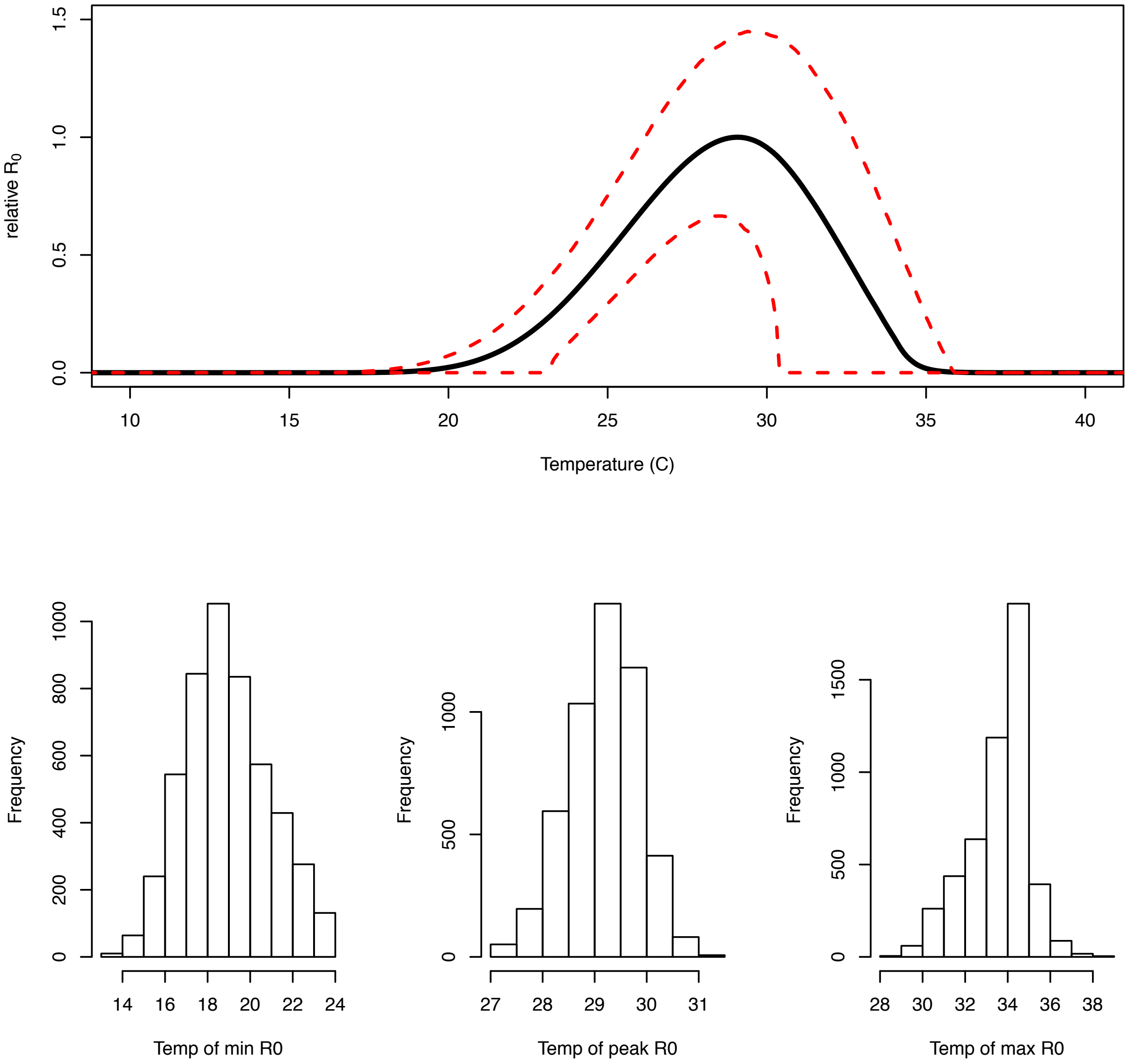

Fig. H

Top, uninformative prior model of *Ae. aegypti* R_0 versus temperature model mean (black line) and 95% highest posterior density intervals (red dashed lines), for constant temperatures. Bottom, histograms of the minimum, maximum, and optimum temperatures for transmission.

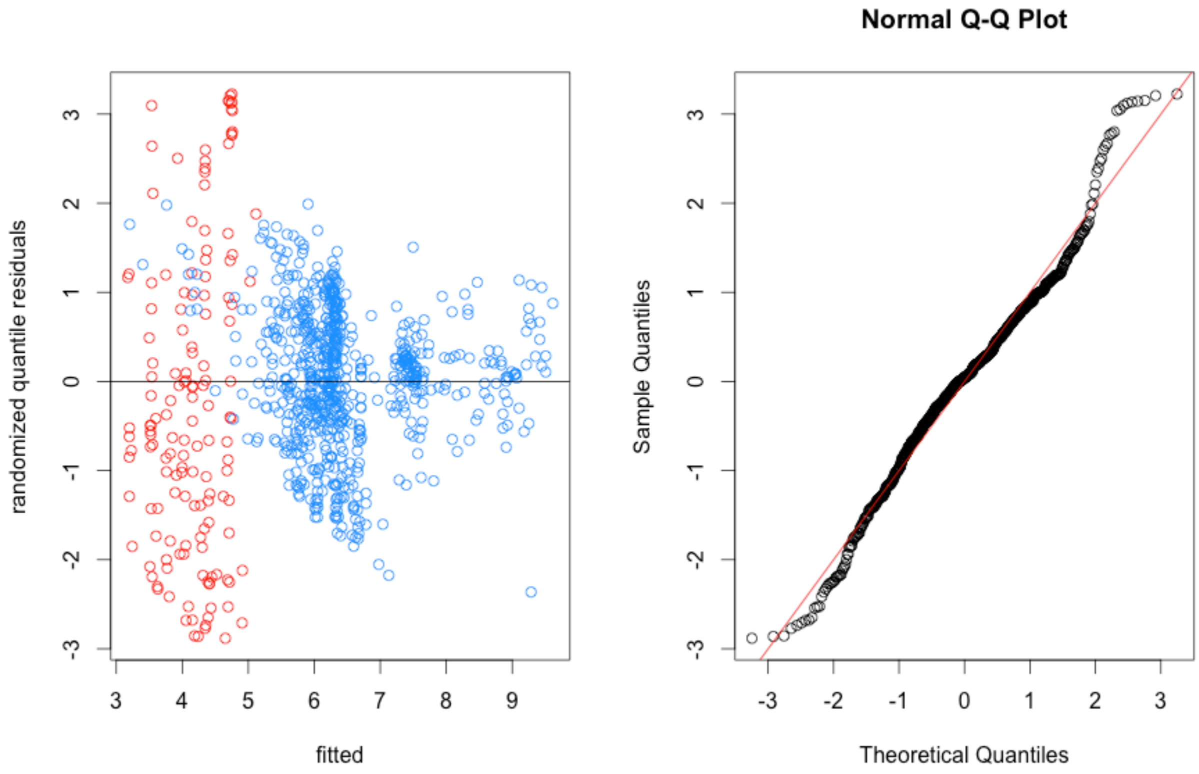

Fig. I

Randomized quantile residuals extracted in R using the *qresid* function in the package *statmod* (7) for the fitted model shown in Fig. 3 of the main text. Randomized Quantile Residuals are interpreted as standard residuals, and should be normally distributed if the assumptions of the underlying model are appropriate for the data. Left: residuals plotted versus the fitted value for DENV (blue) and CHIKV/ZIKV (red). Right: QQ plot for the quantile residuals.

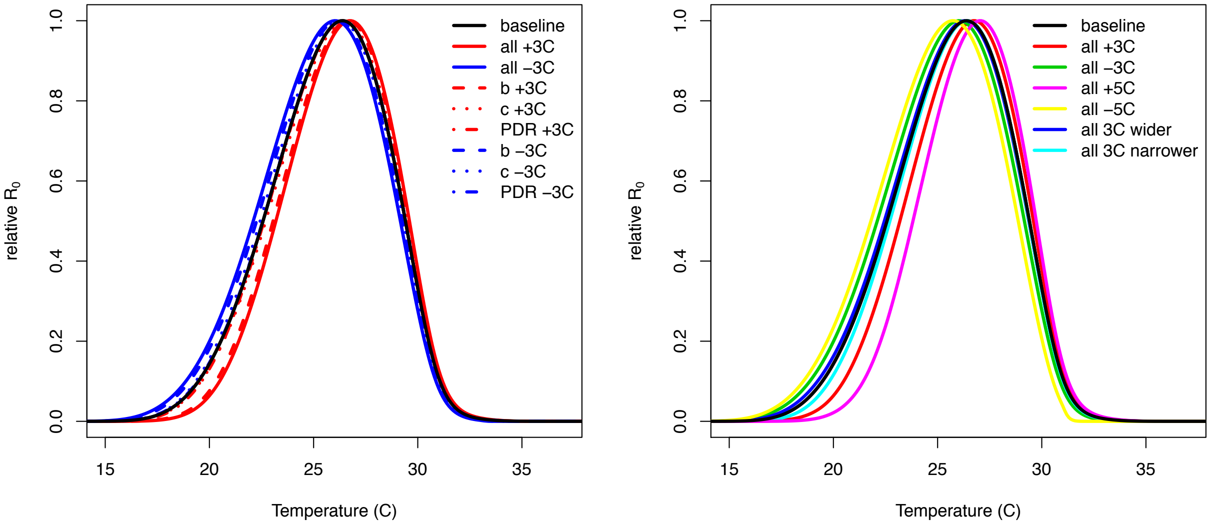

Fig. J

Sensitivity analysis on the *Ae. albopictus* R_0 model at constant temperatures for vector competence (*b* and *c*) and parasite development rate (*PDR* = 1/extrinsic incubation period), in which these traits are shifted individually and together +/-3°C (left panel), or all three are shifted +/-3°C, +/-5°C, or the curves are made 3°C narrower or wider with the same optimum (right panel).

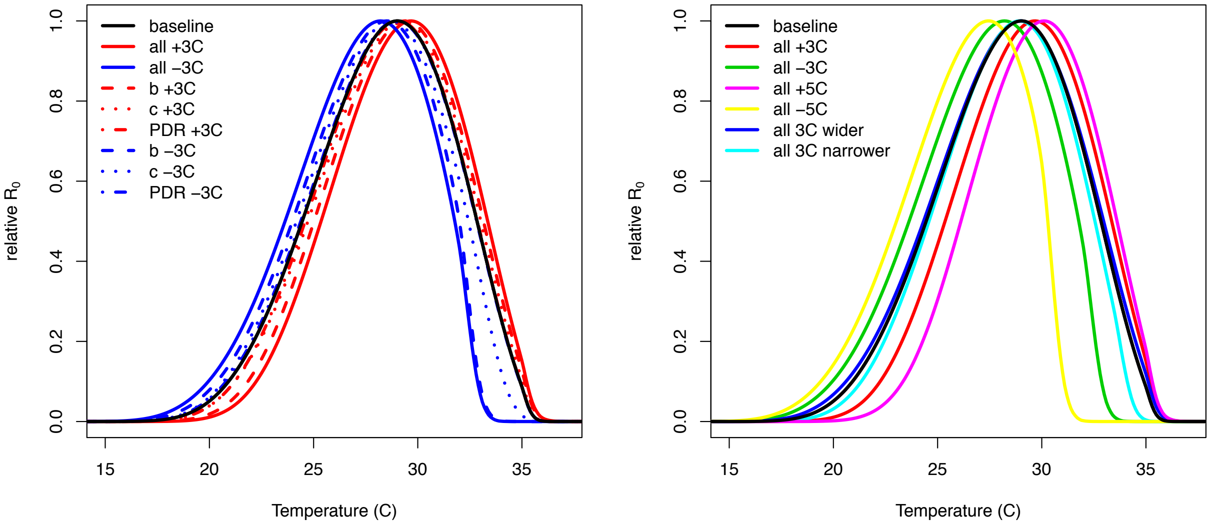

Fig. K

Sensitivity analysis on the *Ae. aegypti* R_0 model at constant temperatures for vector competence (*b* and *c*) and parasite development rate (*PDR* = 1/extrinsic incubation period), in which these traits are shifted individually and together +/-3°C (left panel), or all three are shifted +/-3°C, +/-5°C, or the curves are made 3°C narrower or wider with the same optimum (right panel).

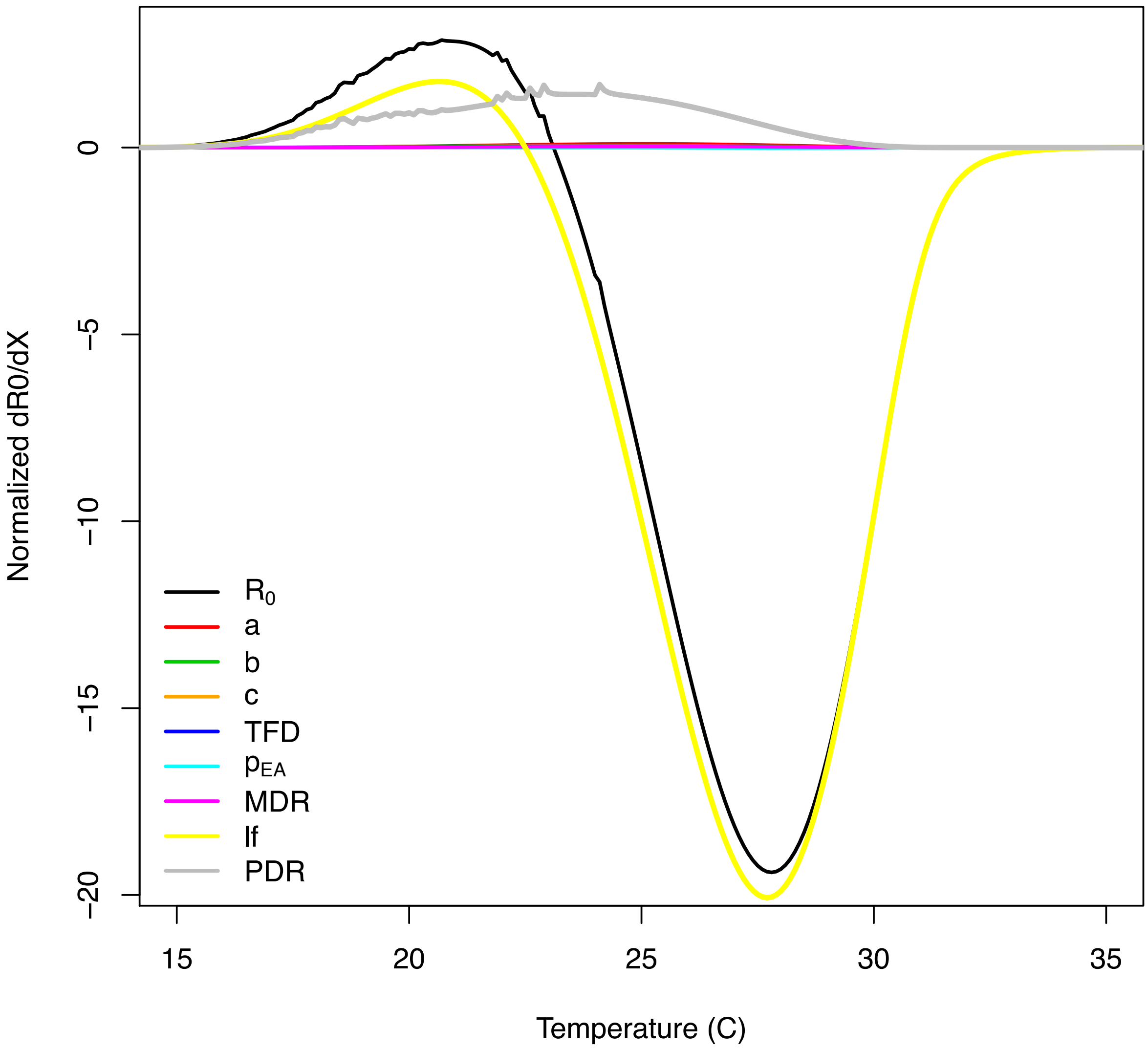

Fig. L

Sensitivity analysis on the *Ae. albopictus* R_0 model, showing the derivative of R_0 with respect to each parameter, divided by R_0, at each temperature. Parasite development rate (PDR) has the largest positive effect on R_0 for most of the temperature range, while mosquito lifespan (lf) has a strong negative effect at warm temperatures. Parameter names are listed in Table A in S2 Text.

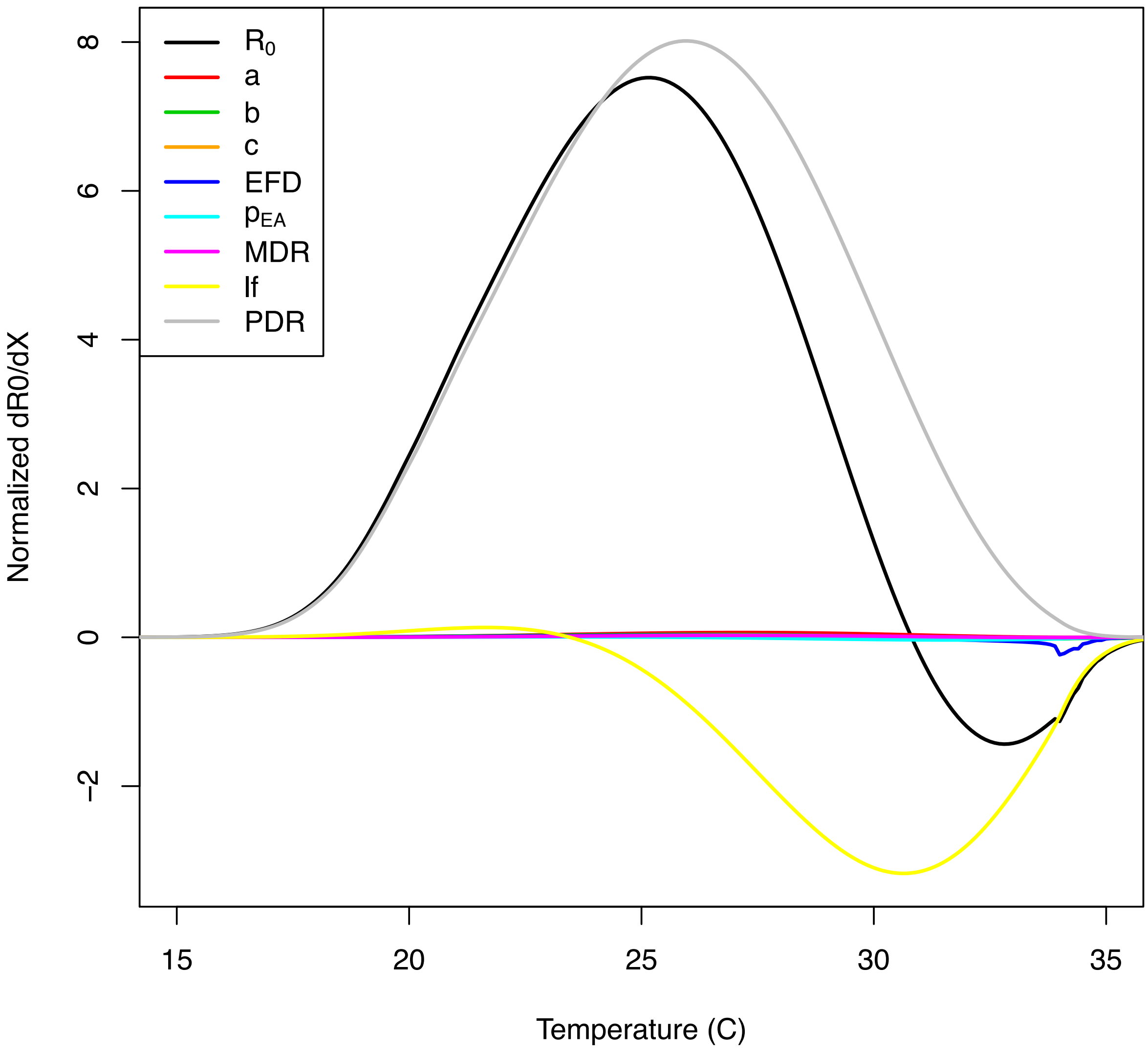

Fig. M

Sensitivity analysis on the *Ae. aegypti* R_0 model, showing the derivative of R_0 with respect to each parameter, divided by R_0, at each temperature. Parasite development rate (PDR) has the largest positive effect and mosquito lifespan (lf) has the largest negative effect on R_0 for most of the temperature range. Parameter names are listed in Table A in S2 Text.

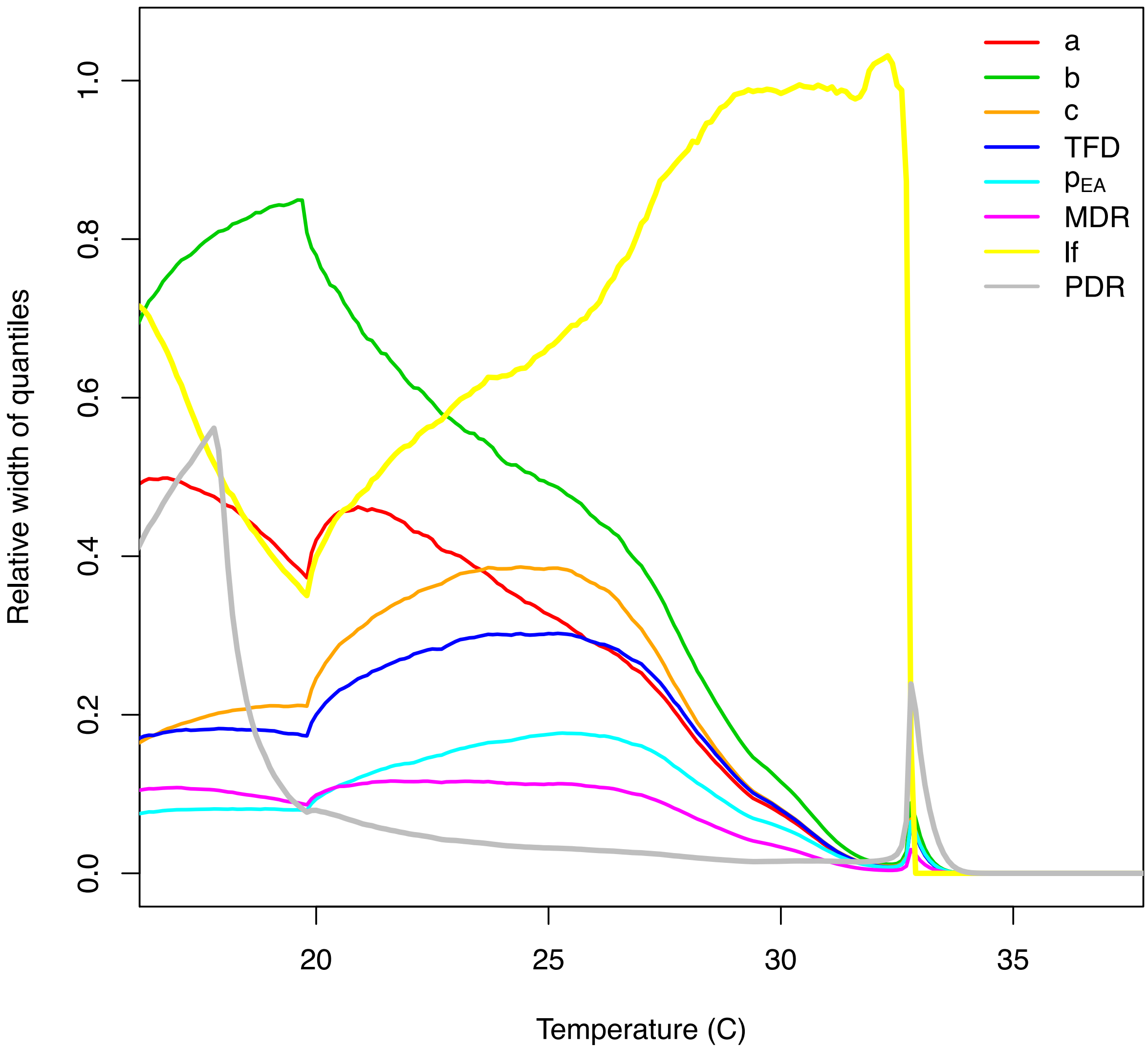

Fig. N

Uncertainty analysis for *Ae. albopictus* R_0 model, showing the relative width of the 95% HPD intervals on R_0 that is due to each parameter, compared to the overall uncertainty. Each line shows the width of the 95% HPD interval on R_0 when calculated using draws from the posterior distribution of the focal parameter and the posterior means of the other parameters, divided by the width of the 95% HPD interval on R_0 when all parameters are drawn from their posterior distribution. This illustrates the degree to which uncertainty in R_0 arises from uncertainty in the component parameters at each temperature value. Mosquito infection probability (b) and lifespan (LF) dominate model uncertainty. Parameters are defined in Table A in S2 Text.

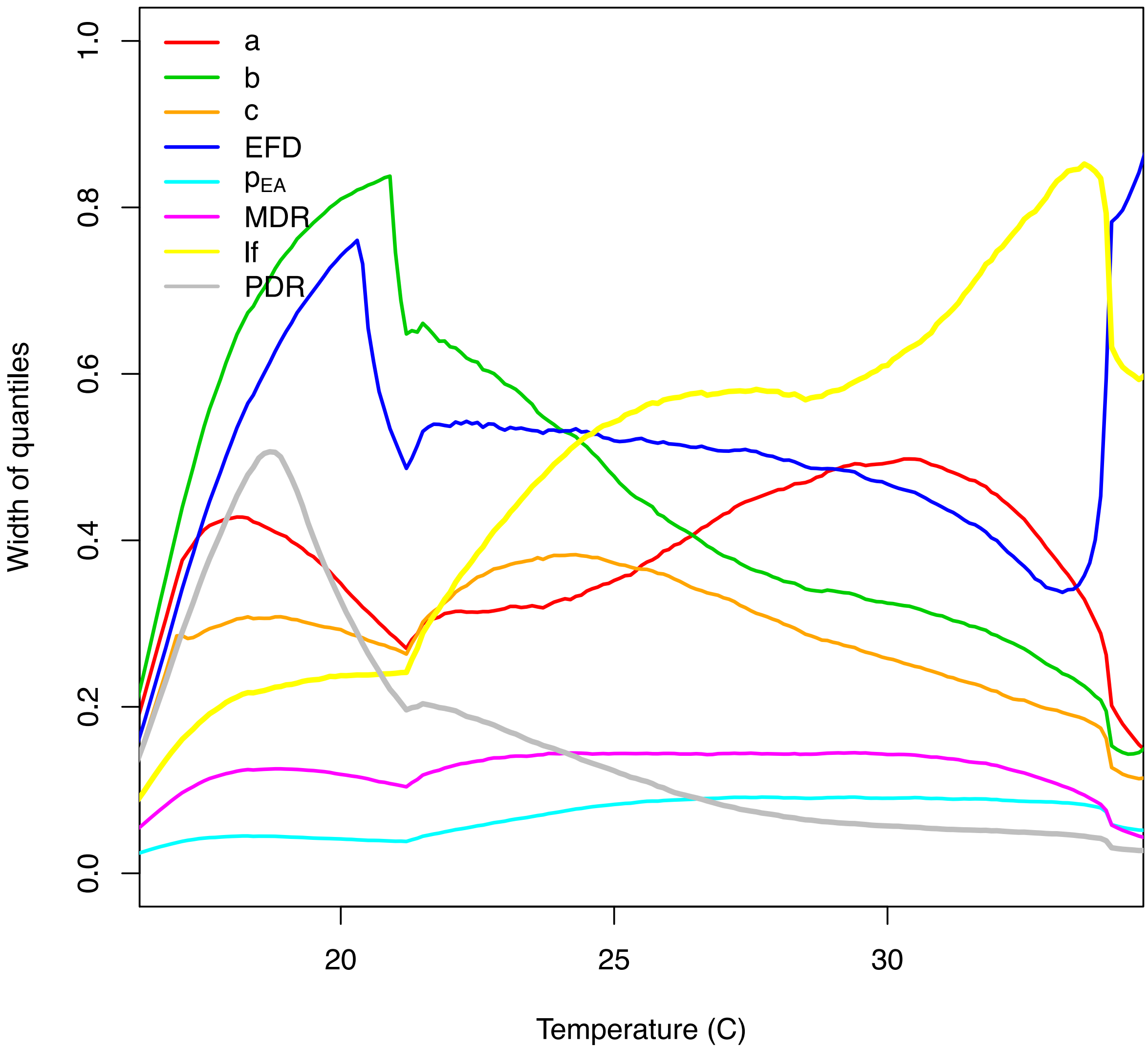

Fig. O

Uncertainty analysis for *Ae. aegypti* R_0 model, as described in the caption for Fig. N. Parameters are defined in Table B in S2 Text.
